# Regulatory Effects of Apatinib in Combination with Piperine on MDM-2 Gene Expression, Glutathione Peroxidase Activity and Nitric Oxide level as Mechanisms of Cytotoxicity in Colorectal Cancer Cells

**DOI:** 10.34172/apb.2022.040

**Published:** 2021-04-03

**Authors:** Mahshid Mohammadian, Zakieh Rostamzadeh Khameneh, Soraya Emamgholizadeh Minaei, Meysam Ebrahimifar, Kosar Esgandari

**Affiliations:** ^1^Department of Medical Laboratory Sciences, Urmia University of Medical Sciences, Urmia, Iran.; ^2^Solid Tumor Research Center, Urmia University of Medical Sciences, Urmia, Iran.; ^3^Department of Medical Physics and Imaging, Urmia University of Medical Sciences, Urmia, Iran.; ^4^Department of Toxicology, Shahreza Azad University, Shahreza, Iran.; ^5^Department of Medical Physics, Urmia University of Medical Sciences, Urmia, Iran.

**Keywords:** Colorectal cancer, Apoptosis, HCT-116 cells, Apatinib, Piperine

## Abstract

***Purpose:*** Apatinib has been utilized in colon cancer therapies but its efficiency and molecularmechanism are not fully understood. Chemotherapy in combination with non-toxic compoundscan be an effective treatment strategy for cancer. Consequently, this study was carried out toevaluate the effects of apatinib and piperine on colorectal cancer (CRC) cell line and theirpotential anti-cancerous mechanisms in vitro.

***Methods:*** The effects of apatinib and piperine on HCT-116 CRC cells were detected byassessing cell viability using MTT assay. The potential cytotoxic mechanisms of apatinib andpiperine were investigated by evaluating MDM-2 gene expression ratio using real-time PCRassay. Moreover, the glutathione peroxidase (GPX) activity and nitric oxide (NO) levels wereassessed by colorimetric assays.

***
Results:*** The proliferation rate of CRC cells decreased by increasing the concentrations ofpiperine or apatinib. When HCT-116 cells were treated with different concentrations of apatinibin combination with piperine, the synergistic effects were observed (combination index < 1).In HCT-116 cells treated with apatinib and piperine at the concentrations of 0.5×IC50 and0.2×IC50, the MDM-2 gene expression was downregulated and NO levels increased comparedto the untreated control cells and related single treatments. In addition, GPX activity significantlydecreased in combination treatment at 0.5×IC50 concentration of both agents versus singletreatments.

***Conclusion:*** Apatinib in combination with piperine could significantly inhibit the growth ofCRC cells. These cytotoxic effects were induced by regulation of MDM-2 gene expression andinhibition of antioxidant marker.

## Introduction

Colorectal cancer (CRC) is the third-largest cancer world width^1,2^ and is related to a high rate of mortality.^1-5^ Recently, clinical studies have been interested in detecting novel integrating targeted treatments and combination chemotherapy regimens.^2^ Combination chemotherapy comes into importance in CRC treatment but drug resistance is inevitable.^3^ In this regard, evaluating new drug combinations can improve the treatment outcomes.^3,5^ Numerous efforts have been conducted for increase understanding of the CRC heterogeneity and propose the most effective-tailored treatment to any affected patient.^6^ Oncogene mutations, the existence of multi-drug resistance, and the intolerable side effects limit the efficiency of treatment. So, new treatment protocols for CRC treatment are instantly required.^7^ Angiogenesis is an important feature of cancer growth and metastasis. Vascular endothelial growth factor (VEGF) binding to vascular endothelial growth factor receptor (VEGFR) induces the vascular endothelial cells proliferation and angiogenesis. Anti-angiogenic drugs show anti-cancer efficiency through blocking the blinding of VEGF and VEGFR. Apatinib mesylate as a micromolecular VEGFR-2 inhibitor that binds to VEGFR-2 and strongly inhibits it, exerts anti-cancer efficacy.^8^ Also, piperine that is a Piperidine alkaloid in black pepper, can inhibit cancer cell growth, but the mechanism of action of piperine is not fully understood.^9^ Piperine has been extensively described to inhibit colon cancer growth by G_1_ arrest in the cell cycle, apoptosis induction, and antitumor activities.^9,10^ In the development of anti-cancer agents, it is imperative to assess the treatment efficacy and also cancer response to chemotherapy.^11-14^ One of the main purposes of cancer therapy is the induction of apoptosis.^15^ Apoptosis is identified as programmed cell death in the damaged and normal tissues.^16^ The apoptosis induction in cancerous cells is considered as the main objective of cancer treatment. Indeed, apoptosis is an important regulatory mechanism of normal cells. Definitely, apoptosis dysregulation can cause uncontrolled cell multiplication. This process is described by a series of definite morphological changes with biochemical features that contain intrinsic and extrinsic pathways through a diverse protein which plays a critical function.^17^

The MDM2 protein encoded through the mouse double minute 2 (*MDM2*) gene, is considered as the negative regulatory factor of the p53 protein and can preserve the p53 signaling pathway stability. MDM2 amplification has been assessed in numerous human cancers, comprising colon cancer.^18^ MDM-2 is an oncogene which its over-expression can exerts transformation in cultured cells.^19^

Otherwise, Nitric oxide (NO) has been revealed to induce apoptosis by post-translational alterations and has an anti-cancer role.^20^ On the other hand, as antioxidant enzymes have a critical function in protection of cells against oxidative stress, so, dysregulation of antioxidant enzymes activity, such as glutathione-peroxidase (GPX), are related to cancer.^21^ So, in this study, we investigated the effects of co-treatments of apatinib and piperine with evaluating the some related molecular mechanism in CRC cells.

## Materials and Methods

### Cell culture

HCT-116 cell line was obtained from the Pasteur Institute in Iran. The culture medium contained 10% fetal bovine serum, 1% penicillin and streptomycin, and Dulbecco’s modified Eagle medium. CRC cells were seeded in 96 well cell culture plate andmaintained at 37*°*Cin a 5*%* carbon dioxide in the incubator.

### Cell proliferation assay

HCT-116 cells were cultured in 96-well plates at a density of 1×10^4^/well. Following incubation at 37 °C and 5% CO2 for 24 hours, CRC cells were exposed to increasing concentrations of apatinib (5, 10, 15, 20, 25, 75, 100 μM) and piperine (10, 20, 30, 40, 50, 100, 150, 200 μM). After 48 h of treatment time, a cell viability kit (Kia Zist, Iran) was used to detect cell proliferation. In this regards, 10 µL MTT reagent was added to each well, and the cells were incubated for 3 h at 37°C and 5% CO2, thesupernatants were discardedand a solubilizer was added to each well. The absorption rate was measured by ELISA Reader at 550 nm. The rate of viable cells was determined by measuring the absorbance. Each test in all treatments was repeated at least three times. The IC_50_ values of each agent were determined based on MTT assay results, dose-response assessment and with Compusyn software (ComboSyn, Inc., Paramus, NJ 07652, USA).

In all combined treatments, the concentrations of 0.50×IC_50_, 0.2×IC_50,_ and 0.1×IC_50_ of both agents were utilized for cell viability assay and all other experimental analysis including GPX activity, MDM-2 gene expression, and NO level assays. The interaction between two therapeutic agents based on Chou^22^ method and Compusyn software (ComboSyn, Inc., Paramus, US) were evaluated, and the combination index (CI) determined. In this regard, the CI < 1, CI > 1, and CI = 1 display synergism, antagonism, and additive effect, respectively.

In addition, the Fraction affected (Fa) amount (indicating the cell fraction that affected by the combination treatment) and Dose reduction index (DRI) were evaluated.

### Real-time polymerase chain reaction (PCR)

Total RNA was extracted from HCT-116 cells (untreated and treated with apatinib and piperine at different concentrations as mentioned above) using an RNA extraction kit based on the kit protocol (GeneAll, South Korea). The First-strand cDNAs were synthesized by GeneAll cDNA synthesis kit (GeneAll, South Korea) and utilized as templates to perform real-time PCR, following the manufacturer’s instruction. The MDM2 gene expression was measured by real-time PCR using a Real Q Plus 2x Master Mix Green (Ampliqon, Denmark). Real time-PCR via cDNAs and specific primers were carried out in annealing temperature at 57°C for 30 seconds. Relative MDM2 expression was normalized to β-actin housekeeping gene and calculated by 2^-ΔΔCt^. Error bars in control and treated groups show the Standard deviation.

### Nitric oxide assay

In order to assay the NO level in untreated and treated cells with various concentrations of apatinib and piperine in single and combined treatments, the cell supernatants were collected carefully. Assessments of NO level in various treatments were performed based on the kit protocol [ZellBio GmbH (Germany)]. The absorbance of each sample was read at 550 nm and NO levels were detected based on a standard curve.

### Glutathione peroxidase activity assay

To evaluate the possible cytotoxic mechanism of apatinib and piperine, the GPX enzyme activity was assessed with the colorimetric method. In this regard, after various treatments as mentioned, cell culture supernatants of each sample were collected carefully, then the GPX activity was determined according to the kit protocol [ZellBio GmbH (Germany]. In the next step, the absorbance at 412 nm was measured and GPX activity was calculated by this formula:

*GPX activity (U/ml)=(OD control-OD sample)/ (OD standard-OD blank) ×6000*

### Statistical analysis

Data were expressed as mean ± standard deviation. All data analysis was performed by one-way ANOVA, followed by the Tukey’s test utilizing GraphPad Prism version 4.0 and SPSS software, V. 10. P-value < 0.05 was considered as significant level.

## Results and Discussion

The cytotoxic effects of apatinib and piperine in HCT-116 CRC cells were detected by MTT assay. The potential mechanisms were investigated by assessing the MDM-2 mRNA expression ratio in vitro using the real-time PCR assay. Moreover, the GPX activity and NO levels were evaluated by colorimetric assays.

**Figure 1:**
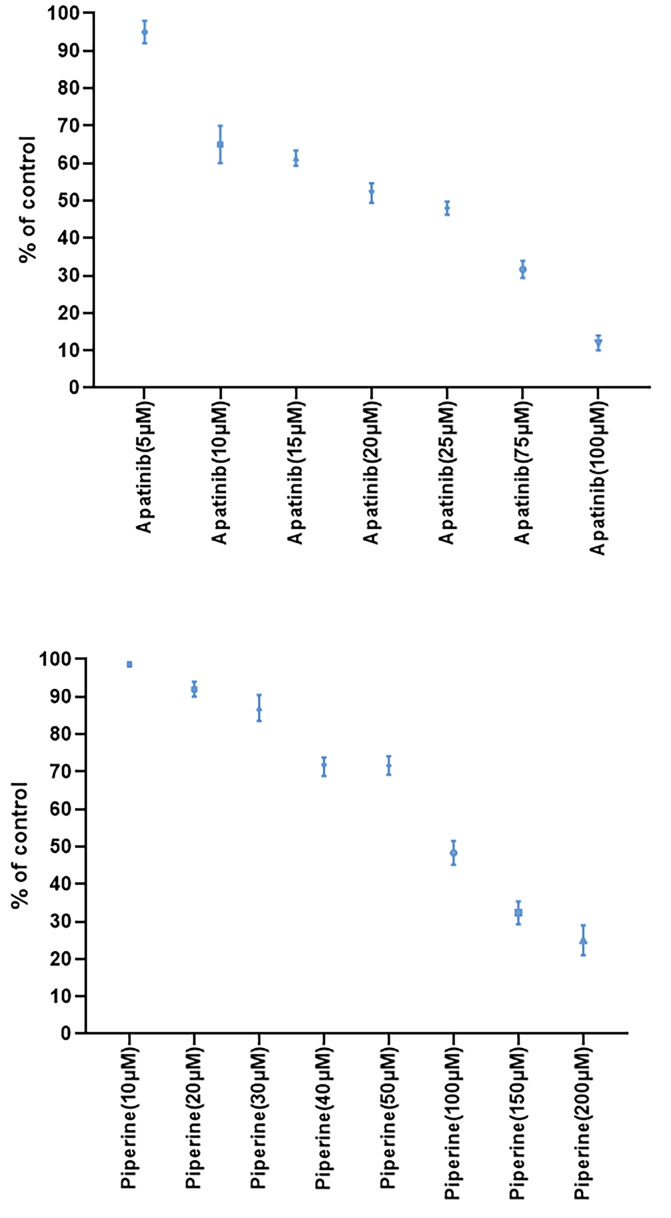
The results of cell viability using MTT assay for Apatinib and Piperine at various concentrations after 48 h treatment. Each value shows the mean ± standard deviation.

In the first step, we evaluated the cytotoxic effects of monotherapies at different concentrations of apatinib or piperine after 48 hours treatment and dose-response assessment were performed. Results of cell viability assays in single therapies were presented in Figure 1. Both agents (apatinib or piperine) in single treatments decreased the cellular viability in a concentration-dependent pattern.

Treatment of HCT-116 cells with increasing concentrations of apatinib or piperine exhibited a reduction in the cellular viability in dose-dependent patterns. The IC_50_ values for apatinib and piperine drugs were equal to 26 and 94 µM, respectively. All combined treatments lead to the synergistic interaction. Indeed, all combined cases showed significant decreased cell viability in comparison with single treatments in same concentrations.

Results of DRI, CI, and Fa for combined treatments at different concentrations were presented in Table 1.

**Table 1:** Results of CI, Fa, and DRI in drug reaction at the various concentrations of Apatinib and Piperine in combined treatments which were measured by Compusyn software. Data showed as mean ± standard deviation.

**Combinations**	**Fa**	**DRI for Apatinib**	**DRI for Piperine**	** CI**
Apatinib-Piperine (0.5×IC_50_)	0.75 ± 0.043	4.52 ± 0.71	3.71 ± 0.45	0.49 ± 0.07 (synergistic effect)
Apatinib-Piperine (0.2×IC_50_)	0.63 ± 0.04	7.57 ± 0.99	6.83 ± 0.68	0.28 ± 0.03 (synergistic effect)
Apatinib-Piperine (0.1×IC_50_)	0.38 ± 0.03	7.13 ± 0.69	7.65 ± 0.57	0.27 ± 0.02 (synergistic effect)

Fa, fraction affected; DRI, dose reduction index; CI, combination index.

**Figure 2:**
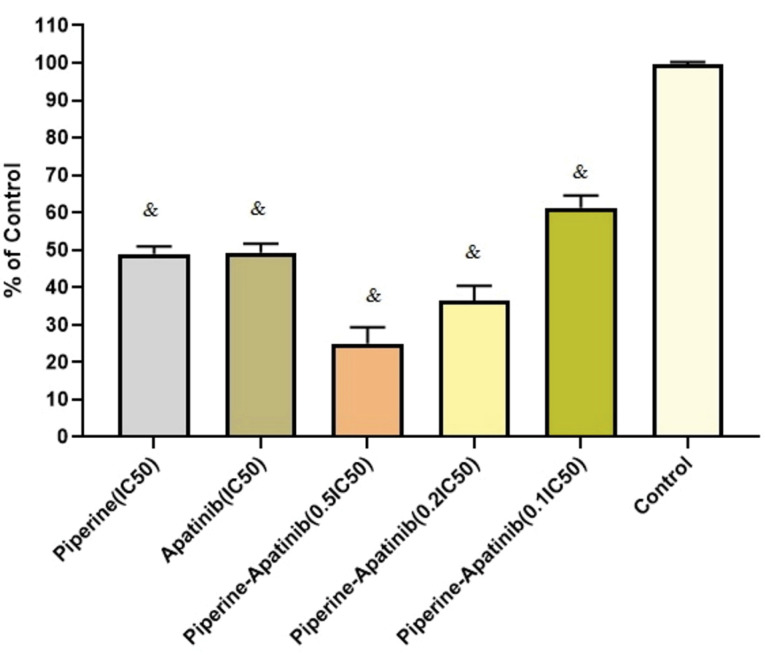
Cellular inhibitory effects of single and combined therapies with apatinib and piperine at different concentrations after 48 h treatment in HCT-116 CRC cells. Each value shows mean ± standard deviation). ^&^ Significant differences compared to untreated control cells (*P* < 0.05).

As presented in Figure 2, after combined treatments with apatinib and piperine, the cell viability in terms of 0.5×IC_50_, 0.2×IC_50,_ and 0.1×IC_50_ concentrations were decreased significantly compared with untreated control cells (*P* < 0.05). In 0.5×IC50 and 0.2×IC50 concentrations in combination treatments, the cell viability were lower than that of corresponding monotherapies including apatinib and piperine in IC_50_ concentrations (*P* < 0.05).

**Figure 3:**
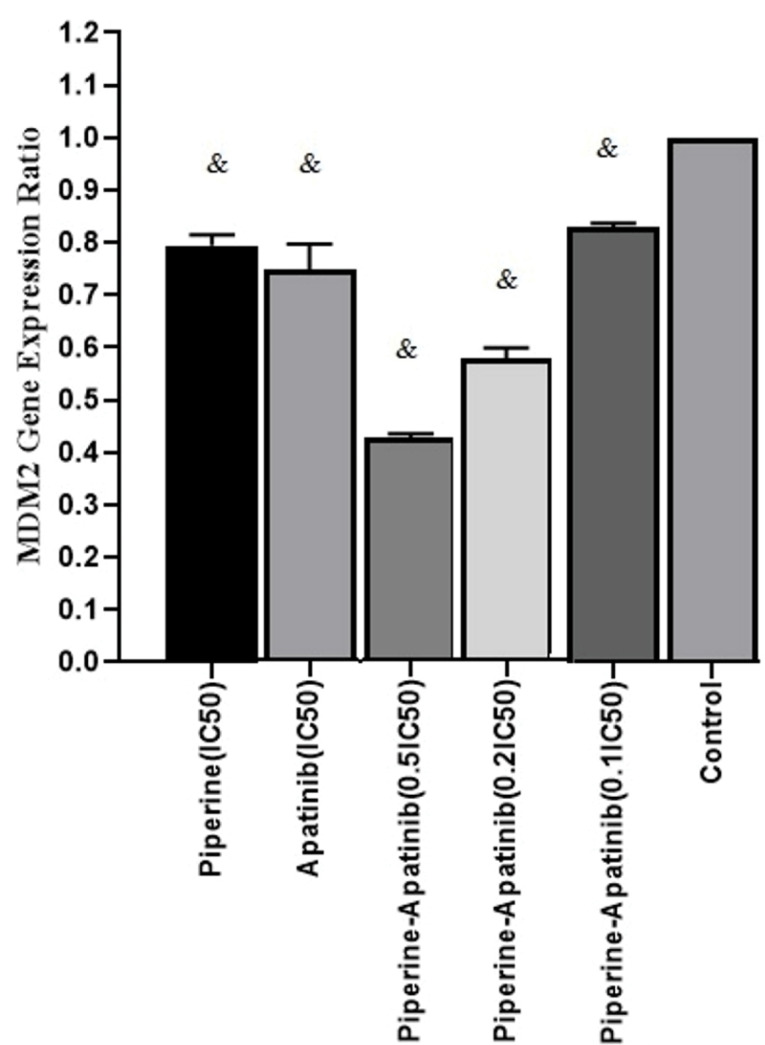
The MDM2 gene expression ratio which was assessed by the real-time PCR method in treated and untreated cells with Apatinib and Piperine after 48 h. each column shows the mean fold change ± standard deviation which was normalized to a housekeeping gene (β-actin) and calculated by 2^-ΔΔCt^.^&^ Significant difference compared to untreated control cells (*P* < 0.05).

In order to study the molecular mechanism of apatinib and piperine effects in CRC cells treated with single and combined drugs, the MDM2 gene expression ratio was evaluated by Real-time PCR assay and normalized to β-Actin as a house-keeping gene. The mean fold changes for each treatment and control cells were calculated by 2^-ΔΔCt^.The results of gene expression levels (Figure 3) showed that the gene expression ratio was decreased in single and combined treatments in compared with control cells (*P* < 0.05). The MDM2 gene expression ratio in combined treatment groups (at concentrations of 0.5×IC_50_ and 0.2×IC_50_) decreased versus single treatments (*P* < 0.05).

**Figure 4:**
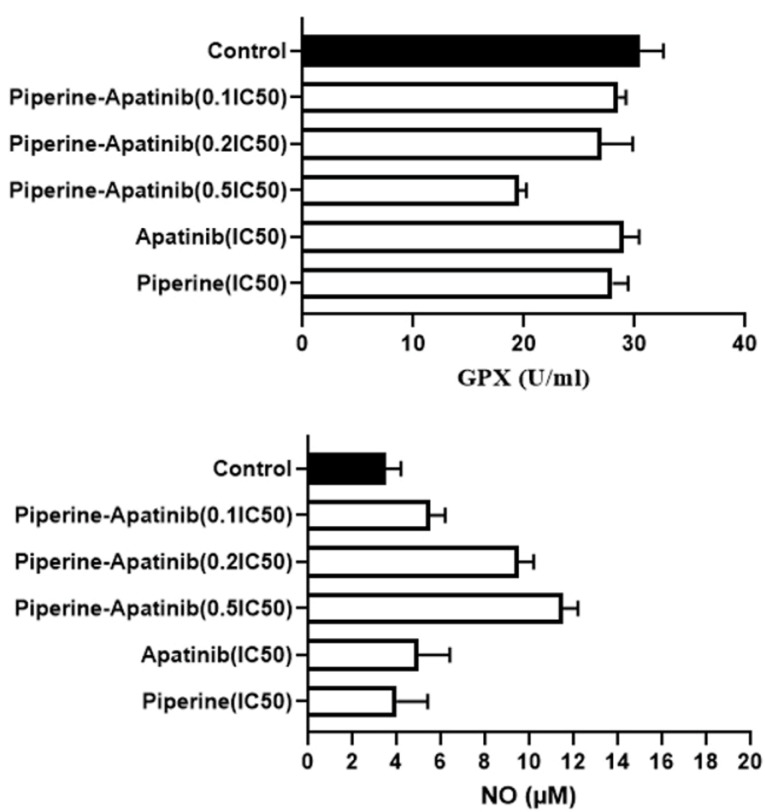
The levels of NO and GPX activity in HCT-116 cells treated with various concentrations of Apatinib and Piperine in single and combination treatments. Data were expressed as mean ± standard deviation.

In this presented study, the GPX activity was evaluated by the colorimetric assay. The results (Figure 4) showed that there was a significant decrease in GPX activity in the combined treatment group at 0.5×IC_50_ concentration of both drugs in comparison with the control group and related single treatments (*P* < 0.05).

The results of the NO assay (Figure 4) showed that the level of NO in the combination groups at the concentrations of 0.5×IC_50_ and 0.2×IC_50_ were more than that of the untreated control group (*P* < 0.05). In addition, there was an elevation in NO levels in combined groups (0.5×IC_50_ and 0.2×IC_50_ concentrations) compared to monotherapies (apatinib and piperine in IC_50_ concentration).

Combination treatments involve the administration of conventional chemotherapeutic agents together with natural bioactives (typically from a plant). The anticancer drug combination may be applied to cancer cell cultures.^23^ Indeed, there are limitations to the effectiveness of several cancer therapies because of the systemic toxicity. Therefore, Chemotherapy with non-toxic compounds can be a strategy for decreasing cancer incidence. Numerous natural agents have showed chemotherapeutic potential.^12^ In this regard; we evaluated the efficacy of apatinib as a chemotherapy drug combined with piperine as a natural agent in the CRC cell line. Our results demonstrated that synergistic effects were observed in the combination treatments of apatinib and piperine at concentrations lower than the IC_50_ values of each agent. Also, the combination treatments regulated the MDM2 gene expression levels and increased NO levels in cell culture, which these effects are related to inducing cytotoxicity. Moreover, combined therapy at the concentration of 0.5×IC_50_ decreased the GPX enzyme activity that indicates the efficacy of this combination treatment and induction of cytotoxicity.

The p53 is the main transcription factor regulating cellular pathways including apoptosis and cell cycle. It acts as a central defense mechanism toward cancer progression and is controlled by interaction with the MDM2 (oncoprotein). The inhibition of MDM2-p53 interaction displayed a striking treatment strategy for cancer therapy.^24^

At the molecular level, piperine can affect numerous effector proteins involved in the apoptosis pathway and can stimulate extrinsic and intrinsic apoptosis process. Piperine repressed the cancer development and metastasis in a cancer model.^25^ In our study, piperine could downregulate the MDM-2 gene expression as an oncogenic mediator.

In a similar study, piperine elevated the anti-proliferative and cytotoxic effects of doxorubicin and paclitaxel in cell line,^26^ which was parallel to our results.

Piperine has elevated the cytotoxicity of paclitaxel and doxorubicin in cell line. Moreover, piperine in combination with doxorubicin and paclitaxel induced P21 expression. These researchers recommended that the molecular mechanism has to be further assessed to recognize the definite function of piperine.^26^ Likewise in another study, the isobologram and the CI of the combination of Paclitaxeland piperineshowed synergistic effects which were in accordance with our results.^27^

Correspondingly, in another study similar to our research, apatinib in combinatorial cases showed the anti-cancer effect. Furthermore, apatinib exhibited synergistic interactions with Paclitaxel plus 5-fluorouracil chemotherapeutic agents*in vivo*.^28^ Also, a related study confirmed that apatinib displayed potentially inhibitory impacts in pancreatic cancerous cells and Astragalus polysaccharide increased the anti-cancer effects of apatinib by decreasing phosphorylation of AKT, and MMP-9.^29^ In addition, a recent study indicated that in AGS cells Astragalus polysaccharide improved the antitumor efficacy of apatinib by inhibition of AKT signaling pathway.^30^

Our study found that piperine or apatinib induced cytotoxicity effects in a dose-dependent manner. Nevertheless, when piperine was combined with apatinib, the expression of the MDM-2 as an oncogene was decreased significantly compared with the apatinib and piperine alone treated groups.

It has been indicated that NO induce apoptosis.^20^ In this regards, piperine and apatinib can increased the NO level in double combinatorial cases including 0.5×IC50 and 0.2×IC50 concentrations versus single therapies.

Therefore, we speculated that apoptosis induction might be associated with the synergetic effects of piperine and apatinib in the present study. Our current study is evidence that indicate pharmacological regulation of the MDM2 gene expression may be related to cytotoxicity, which exerts by these treatments. Piperine in combination with apatinib showed more cytotoxic effects compared to monotherapies by reducing their concentrations in combination treatments. Further studies including clinical trials should be carried out for these new cancer therapies. The combination of apatinib and piperine not only reduced drug concentrations also promoted CRC treatment efficiency.

## Conclusion

Overall, in this investigation, piperine as a natural anti-cancer agent is proving efficacious in combination with apatinib at low concentrations, which could be accounting for possible anti-cancer effects of this combination in CRC cells. Based on our results elevating cytotoxic activity of both agents in the combined treatment group might be related to the increased NO level. Nevertheless, the effects of this combination in cell cycle regulation as well as the decreased expression level ofMDM-2might be examined by further studies.

## Acknowledgments

We are thankful to Mr. Mohammad Aziz Rasouli for his technical support.

## Ethical Issues

This article does not contain any studies with human subjects or animals performed by authors.

## Conflicts of Interest

The authors declared no conflicts of interest.
